# Stable populations and Hardy-Weinberg equilibrium

**DOI:** 10.1186/s41065-023-00284-x

**Published:** 2023-05-05

**Authors:** Alan E. Stark

**Affiliations:** 1 School of Mathematics and Statistics FO7, The University of Sydney, Sydney, NSW 2006 Australia; 2 Balgowlah, Australia

**Keywords:** Autosomal locus, Five alleles, Hardy-Weinberg law, Non-random mating

## Abstract

The conditions on the mating matrix associated with a stable equilibrium are specified for an autosomal locus with five alleles. This points the way to the maintenance of Hardy-Weinberg proportions with non-random mating. The myth of random mating is exposed.

## Introduction

Crow [1] explains the importance of the Hardy-Weinberg principle for population genetical analysis. His final sentence is ‘In this issue of GENETICS, C. C. Li shows that random mating is a sufficient, not a necessary condition for H-W ratios.’ Li [2] coined the term ‘pseudo-random mating’ to apply to his model which demonstrated that Hardy-Weinberg proportions can be maintained with non-random mating for an autosomal locus with two alleles. This fact is implicit in a formula given by Stark [3], as is shown below.

Crow (1999), in discussing the origins of the Hardy-Weinberg law, reproduces the argument of Hardy (1908), including Hardy’s question: ‘in what circumstances will this distribution [$(p + q)^{2} :2(p + q)(q + r):(q + r)^{2}$] be the same as that in the generation before?’ [4, 5]. This paper considers the broader question as to what conditions applied to the matrix of mating proportions ensure that the frequency distribution of the offspring is the same as that of the parents. The Hardy-Weinberg principle is just a special case and, using the broader conditions, the fact that Hardy-Weinberg proportions can be maintained with non-random mating can be justified.

Although the prominent population geneticist C. C. Li [2] showed for an autosomal locus with two alleles that Hardy-Weinberg proportions can be maintained with non-random mating, the genetics community has been slow to acknowledge the fact. The explanation may be the apparent simplicity of the assumption of random mating which is usually made to explain the Hardy-Weinberg principle.

This paper concentrates on the maintenance of the genotypic distribution. Stark [6] shows that Hardy-Weinberg proportions are attainable in one round of non-random mating. Stark [7] reviews some of the steps leading to both of these facets of the law.

Stark and Seneta [8] examine the elusive phrase ‘random mating’ which comes up constantly when appeal is made to the Hardy-Weinberg law. Stark and Seneta [9] describe the fundamental contribution to genetics theory, involving Hardy-Weinberg equilibrium, made by the Russian mathematician S. N. Bernstein.

We believe that, despite the fact that much has been written about the Hardy-Weinberg law, there is still something to be discovered. The topic is introduced through an autosomal locus with two alleles. This is followed by a locus with 5 alleles, then by discussion.

## The matrix of mating proportions for 2 alleles

The features of the mating matrix can be illustrated by the case of an autosomal locus with 2 alleles *A* and *B*. The genotypes are *AA*, *BB*, and *AB*, which are numbered respectively 1, 2, and 3. There are 9 mating combinations and the proportions are arranged in a 3×3 matrix with elements *c*_*ij*_, *i,j* = 1, 2, 3. The matrix is symmetric, that is *c*_*ji*_ = *c*_*ij*_. Elements *c*_*ij*_ are non-negative and their sum is 1. The property that the parental genotype distribution is reproduced in the offspring is obtained by constructing a matrix with *c*_*33*_ = 4*c*_*12*_. Table 1 gives two examples in which the genotypic proportions of the parents are 2/9, 3/9, and 4/9.

**Table 1:** Examples of two mating matrices by which the same parental frequencies are reproduced among offspring (elements to be divided by 1908)

(i)					(ii)				
	AA	BB	AB	Total		*AA*	*BB*	*AB*	Total
*AA*	150	100	174	424	*AA*	140	128	156	424
*BB*	100	262	274	636	*BB*	128	328	180	636
*AB*	174	274	400	848	*AB*	156	180	512	848

The proportion of *AA* offspring is

$$c_{11} + c_{13} + c_{33} /4 = c_{11} + c_{13} + c_{12}$$

that is equal to the proportion of *AA* in parents. A corresponding calculation leads to similar results for types *BB* and *AB*. Inspection of the examples in Table 1 shows that the constraint *c*_*33*_ = 4*c*_*12*_ is flexible, that is the same parental proportions can be reproduced with varying values. There is some freedom in varying *c*_*11*_ provided that other elements satisfy the row and column sums requirements and that of symmetry. But in general there is an uncountable number of possibilities.

## The mating matrix for 5 alleles

When the number of alleles is increased the set of constraints on the elements of the mating matrix to ensure that the genotypic distribution of the parents is reproduced in the offspring is correspondingly enlarged. Stark [10] treats the case for 3 alleles and gives a numerical example of non-random mating with Hardy-Weinberg frequencies [10]. Stark [11] gives the case for 4 alleles.

When the number of alleles is *k,* the number of genotypes is *k*(*k* + 1)/2. As will be seen below, among others, the set of constraints will involve terms containing diagonal elements $c_{k + 1,k + 1}$ to $c_{k(k + 1)/2,k(k + 1)/2}$. This gives freedom to choose various sets of values of these elements. As noted above, this will create options for elements $c_{11}$ to $c_{kk}$, provided that they satisfy the properties of the mating matrix.

The constraints on the matrix of mating proportions for 5 alleles are the following:

$$\begin{array}{*{20}c} {c_{66} = 4c_{12} ;c_{77} = 4c_{13} ;c_{88} = 4c_{14} ;c_{99} = 4c_{15} ;c_{10,10} = 4c_{23} ;c_{11,11} = 4c_{24} } \\ {c_{12,12} = 4c_{25} ;c_{13,13} = 4c_{34} ;c_{14,14} = 4c_{35} ;c_{15,15} = 4c_{45} } \\ {c_{67} = 2c_{1,10} ;c_{68} = 2c_{1,11} ;c_{69} = 2c_{1,12} ;c_{6,10} = 2c_{27} ;c_{6,11} = 2c_{28} ;c_{6,12} = 2c_{29} } \\ {c_{78} = 2c_{1,13} ;c_{79} = 2c_{1,14} ;c_{7,10} = 2c_{36} ;c_{7,13} = 2c_{38} ;c_{7,14} = 2c_{39} } \\ {c_{89} = 2c_{1,15} ;c_{8,11} = 2c_{46} ;c_{8,13} = 2c_{47} ;c_{8,15} = 2c_{49} } \\ {c_{9,12} = 2c_{56} ;c_{9,14} = 2c_{57} ;c_{9,15} = 2c_{58} } \\ {c_{10,11} = 2c_{2,13} ;c_{10,12} = 2c_{2,14} ;c_{10,13} = 2c_{3,11} ;c_{10,14} = 2c_{3,12} } \\ {c_{11,12} = 2c_{2,15} ;c_{11,13} = 2c_{4,10} ;c_{11,15} = 2c_{4,12} } \\ {c_{12,14} = 2c_{5,10} ;c_{12,15} = 2c_{5,11} } \\ {c_{13,14} = 2c_{3,15} ;c_{13,15} = 2c_{4,14} ;c_{14,15} = 2c_{5,13} ;} \\ {c_{6,13} = c_{7,11} = c_{8,10} ;c_{6,14} = c_{7,12} = c_{9,10} ;c_{6,15} = c_{8,12} = c_{9,11} } \\ {c_{7,15} = c_{8,14} = c_{9,13} ;c_{10,15} = c_{11,14} = c_{12,13} } \\ \end{array}$$

The validity of the constraints can be tested by calculating offspring proportions and comparing them with the parental proportions. For example the *AB* offspring frequency is:

$$\begin{array}{*{20}c} {2c_{12} + c_{16} + c_{1,10} + c_{1,11} + c_{1,12} + c_{26} + c_{27} + c_{28} + c_{29} + } \\ { + (c_{66} + c_{67} + c_{68} + c_{69} + c_{6,10} + c_{6,11} + c_{6,12} )/2 + } \\ { + (c_{7,10} + c_{7,11} + c_{7,12} + c_{8,10} + c_{8,11} + c_{8,12} + c_{9,10} + c_{9,11} + c_{9,12} )/2.} \\ \end{array}$$

By substituting the equivalent values from the set of constraints, the *AB* frequency is found to be equal to the sum of the elements of the sixth row of the mating matrix, that is to the frequency of type *AB* in the parents.

## Discussion

Feller ([12], p. 132–136) has a section explaining Hardy [5] which includes the following definition of random mating: ‘If *r* descendants of the first filial generation are chosen at random, then their parents form a random sample of size *r*, with possible repetitions, from the aggregate of all possible parental pairs. In other words, each descendant is to be regarded as the product of a random selection of parents, and all selections are mutually independent’. Feller (p. 134) elaborates:

The genotype of an offspring is the result of four independent random choices. The genotypes of the two parents can be selected in 3 ∙ 3 ways, their genes in 2 ∙ 2 ways. It is fortunately possible to combine two selections and describe the process as one of double selection thus: the paternal and maternal genes are each selected independently and at random from the population of all genes carried by males and females, respectively, of the parental population.

It seems that the explanation above is Feller’s way of interpreting Hardy’s ([5], p. 49) statement: ‘A little mathematics of the multiplication-table type is enough to show that in the next generation the numbers will be as

$$(p + q)^{2} :(p + q)(q + r):(q + r)^{2} ,$$

or as $p_{1} :2q_{1} :r_{1}$, say.’ [13]. Hardy takes a population with a genotypic distribution of whatever kind and produces the Hardy-Weinberg form in one round of ‘random mating’. His next step is directed to the question posed by Punnett in 1908 as to why a dominant character should not replace a recessive (or Hardy’s ([5], p. 49) conundrum: ‘in the absence of counteracting factors, to get three brachydactylous persons to one normal’) [13]. Mr. Udny Yule contributed to the discussion following Punnett’s presentation. Yule introduced the trait brachydactyly as an example of dominance. Punnett ([14], pp. 9–10) describes how this came about and was the reason why Hardy’s solution became ‘Hardy’s law’. Hardy [5] pointed out that if random mating produced Hardy-Weinberg proportions once it could repeat the outcome starting from Hardy-Weinberg form. This he put as the criterion that the distribution obeys the identity $q_{1}^{2} = p_{1} r_{1}$. However, Hardy did not consider whether stability could be achieved in any other way, which is the subject of this paper. Hardy’s criterion $q_{1}^{2} = p_{1} r_{1}$ does not give any insight into the nature of the mating system.

Feller (p. 134) writes: ‘In each of the two selections [a gamete from each parent] an *A*-gene is selected with probability *p*, and, because of the assumed independence, the probability of an offspring being *AA* is *p*^2^ [12]. In countless genetical analyses independence is assumed and often leads to another inference embodied in Feller’s (p. 135) remark: ‘It follows in particular that under conditions of random mating the frequencies of the three genotypes must stand in the ratios $p^{2} :2pq:q^{2}$. This can in turn be used to check the assumption of random mating.’

As noted earlier, when resorting to the notion of random mating to justify the use of the Hardy-Weinberg principle, the appeal is to the apparent simplicity of the concept. But, of course, the mating regime expressed in the elements {c_*ij*_} is subject to the same constraints as those set out above when mating is at random.

In the final section of his paper, Mayo ([15], p. 253) has the following comment:

Li [2], followed and elaborated by Stark [6, 7], showed that panmixia is not the only breeding structure that can yield HW proportions so that panmixia is a sufficient but not a necessary condition for HWE. However, no natural population is known to manifest the other possible breeding structures so that it appears unlikely that they need to be considered in data collection and analysis.

Stark [3] was aware of the fact that Hardy-Weinberg proportions can be maintained by non-random mating. Table 2 gives the algebraic form of such a mating system which is a special case of a general formula given in that article. Hardy-Weinberg proportions are maintained by putting λ = 0 in the following general form.

**Table 2:** Mating proportions reproducing offspring with Hardy-Weinberg frequencies

	*AA*	*BB*	*AB*	Total
*AA*	*q*^2^(*q*^2^ + ν*p*^2^)	*p*^2^*q*^2^(1 + ν)	2*pq*^2^(*q* – ν*p*)	*q*^2^
*BB*	*p*^2^*q*^2^(1 + ν)	*p*^2^(*p*^2^ + ν*q*^2^)	2*p*^2^*q*(*p* – ν*q*)	*p*^2^
*AB*	2*pq*^2^(q – ν*p*)	2*p*^2^*q*(*p* – ν*q*)	4*p*^2^*q*^2^(1 + ν)	2*pq*

In detail, the genotypic proportions are:

$$G_{1} = q^{2} + \lambda pq;\,G_{2} = p^{2} + \lambda pq;\,G_{3} = 2pq(1 - \lambda );q = (2G_{1} + G_{3} )/2;p = 1 - q$$

The mating frequencies are

$$c_{ij} = G_{i} G_{j} (1 + \mu d_{i} d_{j} /S + \nu e_{i} e_{j} /T)$$

where

$$\begin{array}{*{20}c} {\mu = 2\lambda /(1 + \lambda );d_{1} = - 2p;d_{2} = 2q;d_{3} = q - p;e_{1} = - p(1 - \lambda )/(q + \lambda p);} \\ {e_{2} = q(1 - \lambda )/(p + \lambda q);e_{3} = 1;S = 2pq(1 + \lambda );T = pq(1 - \lambda )(1 + \lambda )/((q + \lambda p)(p + \lambda q))} \\ \end{array}$$

The model has the property $c_{33} = 4c_{12}$ so that the parental frequencies are reproduced in offspring. Putting $\nu = 0$ yields mating frequencies usually called random mating but more properly should be called proportionate frequencies.

Edwards ([16], p. 1146) writes: ‘Hardy-Weinberg equilibrium is, of course, a mathematical result of embarrassing simplicity… What is amusing is the irony of so great a mathematician having delivered and published so simple an answer’. Edwards ([16], p. 1149) remarks: ‘That such a slight problem should have found its way to so great a mathematician as Hardy is an example of the social network within and between the colleges of Cambridge.’ It turns out that the problem is not as slight as it appears to Edwards. Perhaps deference to Hardy explains why Feller and countless others have overlooked a basic facet of the problem. Edwards does not cite C. C. Li [2] or Stark [6]. As noted above, Crow ([1], pp. 475–476) comments that random mating is a sufficient, not a necessary, condition for H-W ratios.

Li ([2], pp. 736–737) states: ‘An infinite number of patterns of deviations from random mating exists for autosomal loci that would make a population pseudo-random mating. This could be a contributing factor to the robustness of the Hardy-Weinberg law. The usual “tests for random mating” are actually only tests for random union of gametes which yields the Hardy-Weinberg law, whatever the mating pattern in the population. The situation described in this report makes the study of the mating pattern of a population a worthy subject.’ [2].

The reason why the Hardy-Weinberg model continues to be misunderstood may lie in the confusing terminology used to define it. It is a deterministic model and should be described in terms of proportions rather than probability. However, when ‘random mating’ is introduced it conjures up notions of probability and stochastic models. This confusion is evident in Feller’s struggle to explain the Hardy-Weinberg model.

Diaconis ([17], p. 2) writes: ‘Curiously, Hardy’s most well known work outside mathematics has probabilistic underpinnings. … Hardy’s “back of the envelope” calculation is carefully worded with plenty of sensible caveats’. Between these two sentences Diaconis gives a brief summary of Hardy’s explanation of how Hardy-Weinberg proportions can be generated and maintained. This is an example of how a deterministic model is viewed as a stochastic model. Hardy’s phrase ‘a little mathematics of the multiplication-table type’ is not a statement about probabilities but about proportionate mating frequencies which should not be called ‘random mating’.

Weinberg ([18], p. 378) writes: ‘Ganz anders ist das Verhältnis, wenn man die MENDEL’sche Vererbung unter dem Einfluꞵ der Panmixie betrachtet’. As in Hardy, the notion of random mating is applied to a deterministic outcome. Clearly it will be difficult to reform terminology. Fisher ([19], p. 54) writes: ‘It is well known that if mating were at random the frequencies, *P*, 2*Q* and *R* of the three possible genotypes would be related so that *Q*^2^ = *PR*’.

*Emery’s Elements of Medical Genetics* has a typical demonstration of the Hardy-Weinberg principle [20]. FIG. 7.2 (page 92) has the caption “Punnett’s square showing the frequencies of the different matings in the second generation”. The Hardy-Weinberg frequencies of female and male parents [p^2^, 2pq, q^2^] are displayed on the margins. The mating proportions are simply the products of the marginal frequencies. The demonstration continues to derive offspring frequencies, which are the same as those of the parent, by applying Mendel’s law. “Non-random mating” is listed as a “potentially disturbing factor”.

Hardy indicated how Hardy-Weinberg proportions could be reached and sustained in an idealized setting. One could say that it is an unattainable ideal. He did not pursue the question as to whether there is any way, other than by ‘random mating’, that stationarity can be achieved.

Up to this point there is no appeal to chance which enters the picture when the biologist seeks to apply the principle to a population. Matings occur perhaps by chance and gametes are transmitted at least partly by chance. The probability calculus and statistical theory are useful in deciding whether the Hardy-Weinberg model can be used for analytical and predictive purposes.

Weinberg found the model useful for studying the human propensity to produce twins. An account of his life and work is given by Sperlich and Früh [21].
